# Effect of Dissolved Oxygen and Immersion Time on the Corrosion Behaviour of Mild Steel in Bicarbonate/Chloride Solution

**DOI:** 10.3390/ma9090748

**Published:** 2016-09-01

**Authors:** Gaius Debi Eyu, Geoffrey Will, Willem Dekkers, Jennifer MacLeod

**Affiliations:** Department of Chemistry, Physics and Mechanical Engineering, Queensland University of Technology, Brisbane QLD 4000, Australia; g.will@qut.edu.au (G.W.); w.dekkers@qut.edu.au (W.D.); jennifer.macleod@qut.edu.au (J.M.)

**Keywords:** mild steel, bicarbonate, polarization, passivation

## Abstract

The electrochemical behavior of mild steel in bicarbonate solution at different dissolved oxygen (DO) concentrations and immersion times has been studied under dynamic conditions using electrochemical techniques. The results show that both DO and immersion times influence the morphology of the corrosion products. In comparative tests, the corrosion rate was systematically found to be lower in solutions with lower DO, lower HCO_3_^−^ concentrations and longer immersion time. The SEM analyses reveal that the iron dissolution rate was more severe in solutions containing higher DO. The decrease in corrosion rate can be attributed to the formation of a passive layer containing mainly α-FeO (OH) and (γ-Fe_2_O_3_/Fe_3_O_4_) as confirmed by the X-ray diffractometry (XRD) and X-ray photoelectron spectroscopy (XPS). Passivation of mild steel is evident in electrochemical test at ≈ −600 mV_SCE_ at pH ≥8 in dearated (≤0.8 ppm DO) chloride bicarbonate solution under dynamic conditions.

## 1. Introduction

Bicarbonate plays a major role in the dissolution reactions of steel pipelines [1]. Studies indicate that increasing HCO_3_^−^ concentration exacerbates iron dissolution [2,3,4]. To date, the role of bicarbonate ions in the corrosion process of carbon steel is still debated [5] Moreno et al. [6] confirmed that high bicarbonate increased localized corrosion contributing to stress corrosion cracking [7,8] and pitting corrosion [9] in carbon steel pipelines. Thomas and Davies [10] reported that bicarbonate ions reduce the passive-active (Flade) potential for ferric oxides, while increasing the passive-active transition potential for magnetite with increasing HCO_3_^−^ concentration when the concentration is greater than 10^−2^ M. However, some researchers argued that a stable-film formation (passivity) can be achieved when a sufficient amount of bicarbonate is present [11,12,13]. The formation and stability of such passive films depends to a large extent on environmental conditions, such as; applied potential, pH, oxygen, solution chemistry, temperature, fluid flow rate, immersion time and metallurgy of the steel [14,15,16]. Bicarbonate-induced corrosion is potentially relevant in many applications, such as wellbore systems for geological sequestration of CO_2_ [17]. Intergranular stress corrosion cracking (IGSCC) in underground pipelines at potential range (≈−0.625 V_SCE_ to ≈−0.425 V_SCE_) has been linked with the presence HCO3^−^ and CO_3_^2−^ ions at pH (8–10.5) [18]. Delanty and O’Beirne [19] cited a Canadian investigation that revealed that stress corrosion cracking (SCC) in carbon steel X65 was more severe in oxygen restricted areas, presumably because oxygen reduction prevents hydrogen evolution which is thought to facilitate cracking. In contrast, Yunovich et al. reported that SCC susceptibility of API X52 carbon steel was higher in an aerated conditions than in deaerated solution at pH 8 and 10 [18]. Dissolved oxygen has been shown to increase corrosion rates [20,21,22,23,24] as expected however, Beckett et al. and Sarin et al. [25,26] claimed it decreased with higher DO under stagnant conditions. Dissolved oxygen is an electron acceptor in the corrosion of iron as well as oxidation of ferrous iron [27]:

Fe + ½ O_2_ + H_2_O ↔ Fe^2+^ + 2OH^−^ (1)

Fe^2+^ + ¼ O_2_ + ½ H_2_O + 2OH^−^ ↔ Fe(OH)_3 (s)_ (2)

Kuch [28] reported that in the absence of oxygen, it is possible for ferric scale γ-FeOOH (lepidocrosite) previously formed on the metal surface to act as an electron acceptor:

Fe + 2FeOOH _(scale)_ + 2H^+^ ↔ 3Fe^2+^ + 4OH (3)

The corrosion process continues even after DO is depleted according to the *Kuch mechanism*. However, in the absence of lepidocrosite in the scale, the corrosion reaction still proceeds in deaerated water as reported elsewhere [26], indicating that in a near-neutral pH environment, the Kuch mechanism is not the only mechanism of metal loss in deaerated conditions. Baek et al. [29] concluded that DO facilitates the formation of different iron oxide products. For example, FeOOH and γ-Fe_2_O_3_ were formed at high DO concentrations whereas α-Fe_2_O_3_ was formed in deaerated solution at a more positive potential. The influence of dissolved oxygen and immersion time in the corrosion process of mild steel in a near-neutral pH in corrosive media remains controversial. Here, we present an investigation of the corrosion behaviour of mild steel in chloride-containing bicarbonate solutions of varied DO and immersion time in near-neutral pH, under dynamic conditions at moderate temperature (23 ± 1 °C). Potentiodynamic polarization and electrochemical impedance spectroscopy were used to study the corrosion processes for the electrochemical tests. By incorporating a range of surface analysis techniques, including X-ray diffraction (XRD) and scanning electron microscopy (SEM), we were able to characterize the chemistry and morphology of the corrosion products.

## 2. Results and Discussion

### 2.1. Bicarbonate Concentration

**Figure 1:**
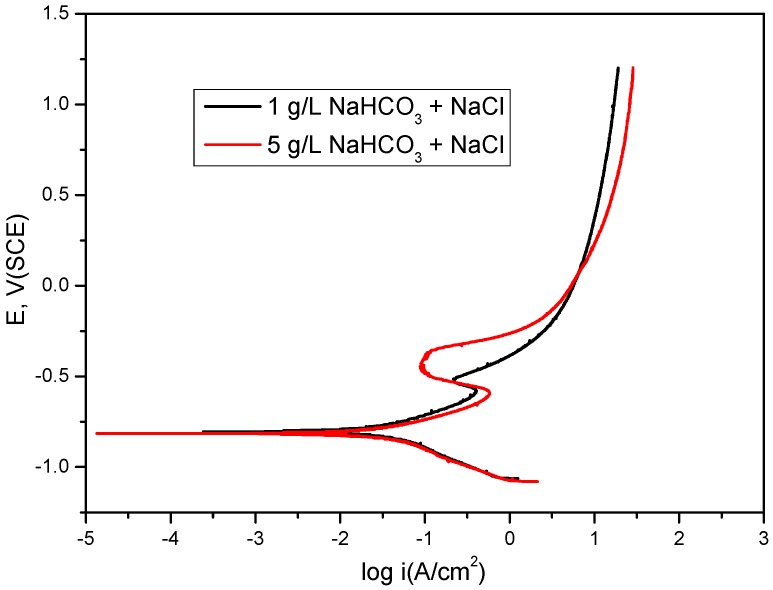
Potentiodynamic polarization for mild steel in 1 g/L and 5 g/L bicarbonate/chloride solutions at 2000 rpm.

Figure 1 shows the polarization profile for mild steel in 1 g/L and 5 g/L bicarbonate with 2 g/L chloride concentration under dynamic conditions. Bicarbonate has a distinct effect on the polarization characteristics in bicarbonate/chloride solution. The dissolution in the active and prepassive region is accelerated by the presence of bicarbonate ions due to the formation of soluble complex anion Fe(CO_3_)_2_^2−^ [3]. At ≈−600 mV_SCE_, Fe(OH)_2_ is converted to Fe_3_O_4_ [29] in dearated bicarbonate solution, resulting in passivation. At this potential (≈−600 mV_SCE_), Fe_2_O_3_/Fe_3_O_4_ forms an oxide film at pH ≥ 8,. The anodic polarization current was greater with 5 g/L bicarbonate solution at corrosion potential ≈−600 mV_SCE_. However, above this potential, the dissolution decreased with increasing bicarbonate concentration.

**Figure 2:**
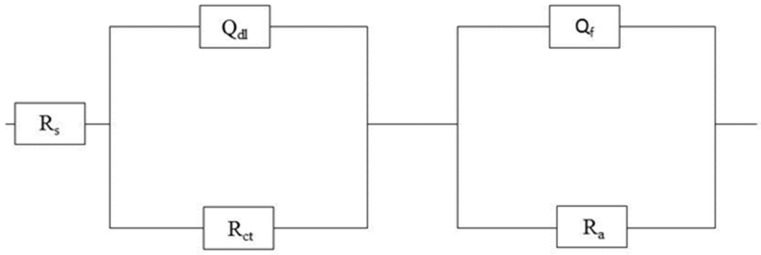
Equivalent circuit for impedance measurements.

The impedance spectra were obtained from a rotating disc electrode (specimen) immersed in solutions containing 1 g/L and 5 g/L bicarbonate in chhloride solution. The data was fit with equivalent circuit shown in Figure 2, and shows a good fit to the data and represents the physical situation expected [30,31], where R_s_, R_ct_, R_a_, Q_dl_ and Q_f_ represent the solution resistance, charge transfer resistance (metal/film interface), adsorption resistance (solution/film interface), double layer capacitance and passive film capacitance, respectively. A constant phase element (CPE) defined by the values of n and Q, is commonly used to compensate for inhomogeneity of electrode surface. The impedance Z, of the constance phase element as given in [32]:

Z_CPE_ = Q^−1^(j*ω*)^−n^, (4)

where Q and n are constant and exponent, respectively, j = ($\sqrt{-1}$) and ω=2πf is the angular frequency in rad/s calculated using *f*, the frequency in H_z_. At low frequency, the impedance and phase angle describe the kinetic response for the charge transfer activity, while at high frequency they depend on surface layer inhomogeneity [33].

**Figure 3:**
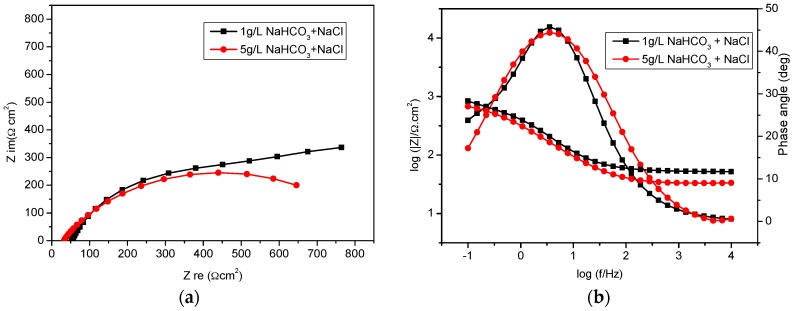
Impedance spectra for mild steel in bicarbonate chloride solution: (**a**) Nyquist plots and (**b**) Bode phase angle and impedance magnitude vs. frequency plots.

**Figure 4:**
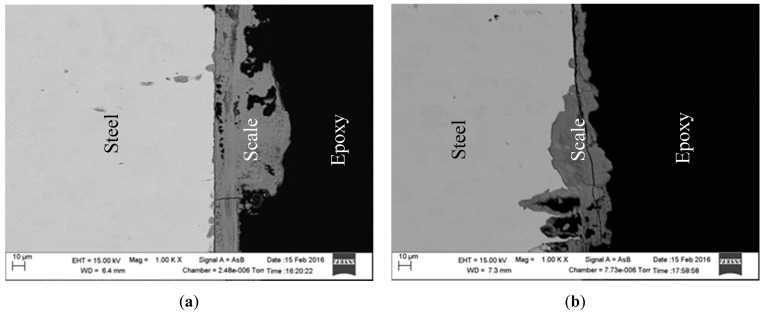
Cross-section of the scale formed at (**a**) 1 g/L (**b**) 5 g/L sodium bicarbonate/chloride solution at 2000 rpm.

Figure 3a,b show the Nyquist and Bode-phase plots measured for specimens in 1 g/L and 5 g/L bicarbonate/ chloride solutions. The impedance spectra show similar trends, although the capacitive loop was depressed with higher bicarbonate concentration. The decrease in the impedance spectra is linked to iron dissolution which contributes to uneven surface roughness and irregular distribution of current density on the specimen surface. The Bode impedance plots in Figure 3b show that at low frequencies, the impedance is slightly lower with higher bicarbonate concentration. The lower bicarbonate concentration exhibits higher impedance in the capacitive region, which implies that a more protective oxide layer is achieved in low concentration bicarbonate. For this lower concentration solution, the phase angle (θ) is higher at low frequencies compared to solution with higher bicarbonate, indicating a more resistive barrier film layer at lower concentration. The cross-sections of the corrosion products shown in Figure 4 reveal that a more uniform corrosion product was formed in 1 g/L bicarbonate in comparison with 5 g/L bicarbonate solution.

### 2.2. Dissolved Oxygen

**Figure 5:**
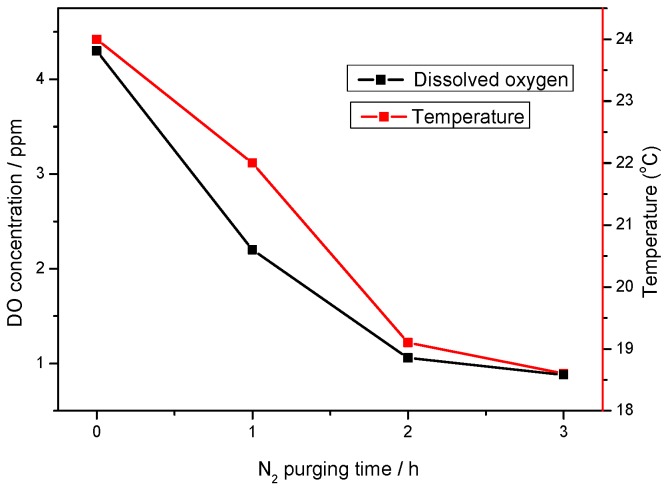
Dissolved oxygen (DO) concentration and temperature as a function of N_2_ purging time.

Dissolved oxygen concentration decreases with nitrogen purging time. In the present experiments, the nitrogen purging also led to a corresponding decrease in temperature as depicted in Figure 5. The test solution temperature was ≈24 °C at 0 h purging, with DO ≈ 4 ppm. The DO decreased significantly to ≈0.8 ppm after 3 h, at which point the temperature decreased to ≈18.6 °C. The decrease in temperature with purging time was due to the cooling effect of the cooler nitrogen gas.

**Figure 6:**
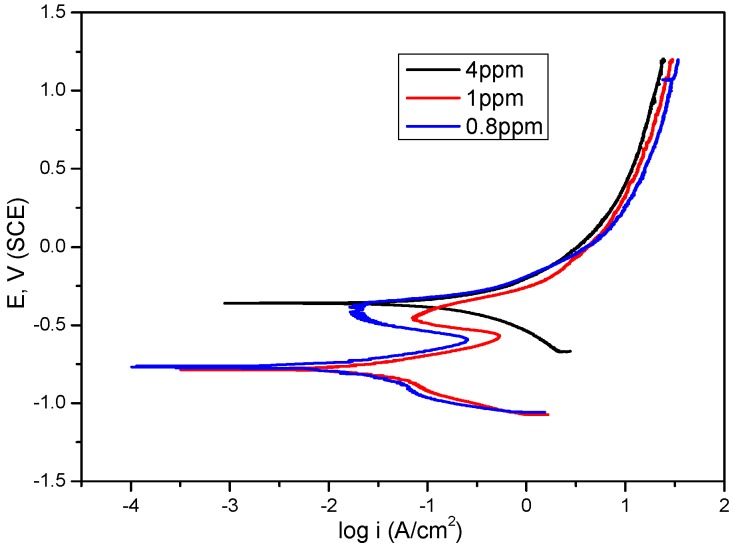
Potentiodynamic polarization of mild steel in 5 g/L sodium bicarbonate /chloride solution in different dissolved oxygen concentrations at 2000 rpm.

Figure 6 shows the polarization curves of mild steel in bicarbonate/chloride solution in different DO concentrations at 2000 rpm. The anodic polarization increased without any retardation at a DO concentration of 4 ppm, whereas one anodic peak appeared in solutions containing 1 ppm and 0.8 ppm DO. The anodic polarization current increases with corrosion potentials below ≈−600 mV_SCE_, indicating iron dissolution while at potentials greater than ≈ −600 mV_SCE_, the anodic current decreases indicating some form of passivation. A potential cause of this behaviour would be as a result of corrosion products. For example, Fe(OH)_2_ formed on the specimen surface, according to the following reaction:

Fe + 2H_2_O → Fe(OH)_2_ + 2H^+^ + 2e^−^. (5)

Fe(OH)_2_ forms a defective hydrous film barrier layer between the specimen and the solution, and consequently retards the anodic current density [34,35]. The corrosion current density decreased with decreasing DO concentration from 201.5 µA·cm^−2^ to 22.3 µA·cm^−2^ at 4 ppm and 0.8 ppm, respectively, as shown in Table 1. This indicates that a more compact and stable oxide film was formed at ≈−600 mV_SCE_ at lower DO. The increase in anodic polarisation current with decreasing DO at a more noble potential could be attributed to the solubility of corrosion product as temperature decreases due to the cooling effect of N_2_ gas and insufficient oxidation at the steel surface to form oxide film other than through the *Kuch* mechanism.

**Figure 7:**
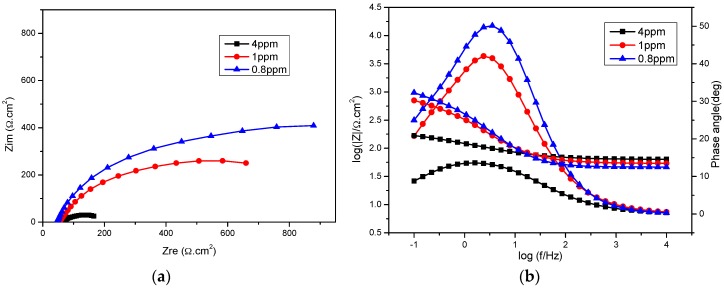
Impedance spectra for mild steel in 5 g/L bicarbonate/chloride solution in different dissolved oxygen concentration at 2000 rpm (**a**) Nyquist impedance plots (**b**) Bode and phase angle plots.

**Figure 8:**
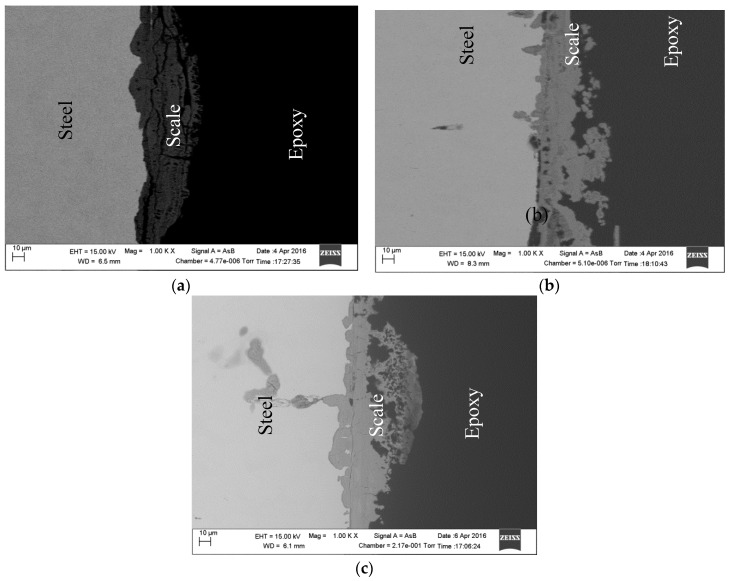
Cross-section of the scale formed at in 5 g/L bicarbonate/chloride solution for (**a**) 4 ppm (**b**) 1 ppm (**c**) 0.8 ppm dissolved oxygen at 2000 rpm.

Figure 7a,b show the Nyquist and Bode-phase angle plots for different DO concentrations in bicarbonate/chloride solution. The impedance semi-circle increases with decreasing DO, which implies that decreasing DO facilitates the formation of a more stable oxide layer at the steel surface. A similar trend can be seen in the Bode impedance magnitude spectra (Figure 7b), where the impedance increases with decreasing DO concentrations at low frequencies. The phase angle shows a higher peak as DO concentration decreases. A higher phase angle value at low frequency indicates a higher surface resistance at the steel. The increase in R_ct_ and a decrease in Q_dl_ with increasing DO from 1015 Ω·cm^2^ at 0.8 ppm to 104.7 Ω·cm^2^ at 4 ppm as shown in Table 2 can be attributed to oxide film formation. The film resistance (R_a_) increases significantly with decreasing DO concentration, suggesting that the corrosion product at the steel surface is more dense and protective at a relatively low DO as shown in Figure 8.

### 2.3. Immersion Time

**Figure 9:**
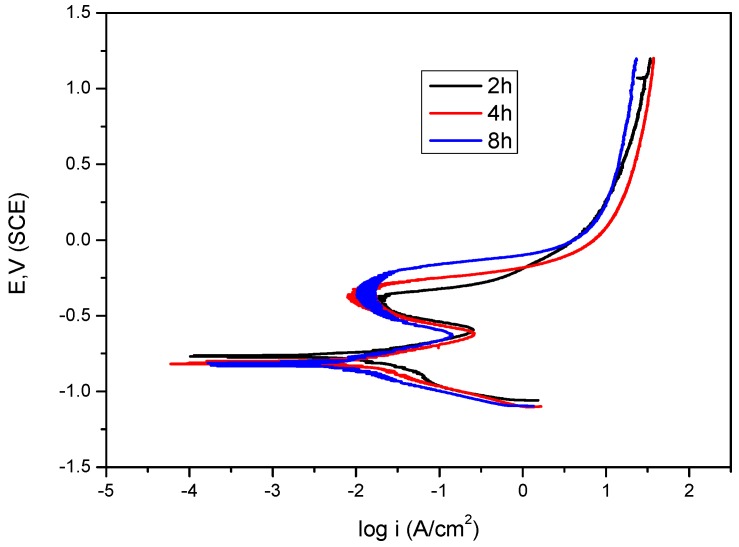
Potentiodynamic polarization for mild steel in 5 g/L bicarbonate/chloride solution at different immersion times at 2000 rpm.

Figure 9 contains polarization curves showing the effect of immersion time on mild steel in bicarbonate/chloride solution at 0.8 ppm DO under dynamic conditions (2000 rpm) rotation speeds. The polarisation curves show that immersion time has a marked effect on anodic and cathodic polarization current. However, the effect was more apparent at corrosion potentials between (−250 and + 250 mV_SCE_), where anodic potential current decreased with immersion time. The curve for each immersion time contains one anodic peak and, as expected, corrosion products on the steel surface become thicker and more compact with immersion time. Siderite can form in relatively low flow velocities at pH > 5, and the film thickness increases with time [16]. The passivation potential range becomes broader with increasing immersion time, with the pitting breakdown potential E_b_ increasing from ≈−355 mV_SCE_ at 2 h to ≈−229 mV_SCE_ after 8 h immersion time as shown in Table 3.

**Figure 10:**
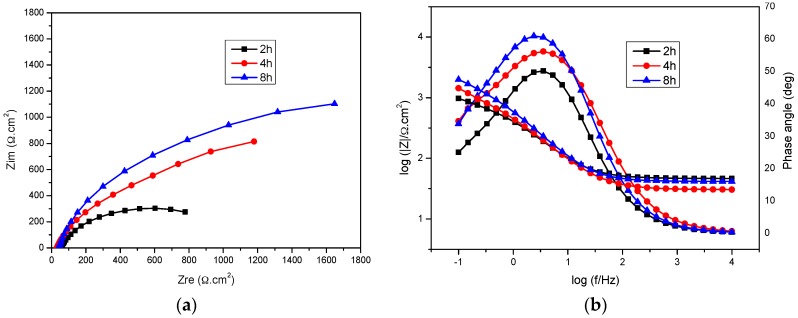
Impedance spectra for mild steel in 5 g/L bicarbonate/chloride solution at different immersion times at 2000 rpm (**a**) Nyquist impedance plots (**b**) Bode and phase angle plots.

**Figure 11:**
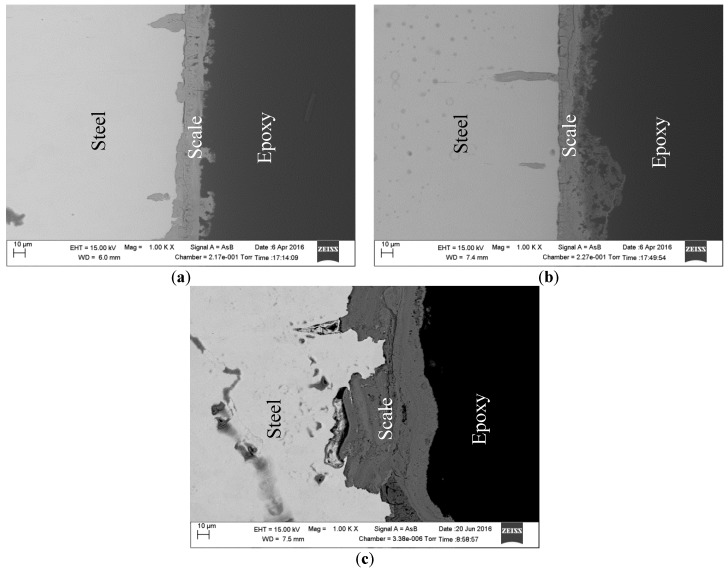
Cross-section of the scale formed in 5 g/L sodium bicarbonate/chloride solution at 2000 rpm after (**a**) 2 h (**b**) 4 h and (**c**) 8 h immersion times.

Figure 10 shows the Nyquist plots and Bode phase angle plots of mild steel in bicarbonate/chloride solution at 0.8 ppm DO at different immersion times at 2000 rpm. The Nyquist plots in Figure 10a show that the diameter of the semi-circle increases with immersion time, which implies that the steel surface resistance improves with immersion time. The result is consistent with more corrosion product being deposited on the the steel surface, and forming a relatively inert surface barrier at the steel–solution interface. The charge transfer resistance (R_ct_) increased from 1015 Ω·cm^2^ to 2700 Ω·cm^2^ in going from 2 h to 8 h immersion time as shown in Table 4, presumably as a result of deterioration of the oxide film on the steel surface. A similar result was reported in our previous work [31]. The Bode impedance magnitude plots show similar trends. The impedance magnitude and the phase angle increase with immersion time as evident in Bode plots at low frequencies as shown in Figure 10b, implying that the corrosion product at the steel surface becomes denser and more protective with immersion time, as is evident in Figure 11. It would therefore be expected that metal dissolution would be retarded, in agreement with the data obtained from polarization curves.

### 2.4. Surface Analysis

**Figure 12:**
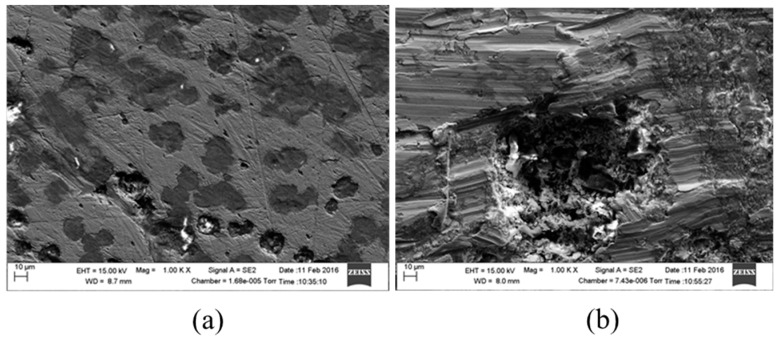
Scanning Electron Microscopy micrographs of mild steel in 5 g/L bicarbonate/chloride solution at 2000 rpm: (**a**) 1 g/L and (**b**) 5 g/L bicarbonate solution.

**Figure 13:**
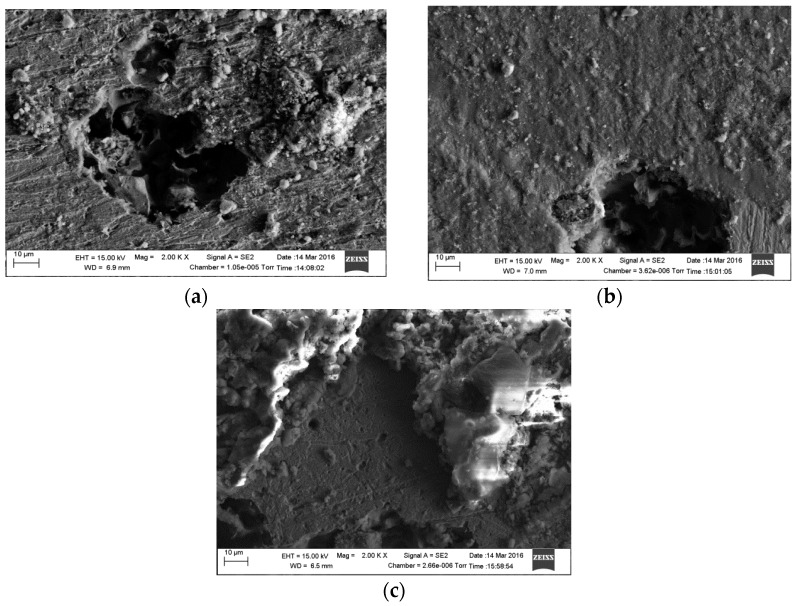
Scanning Electron Microscopy micrograph in 5 g/L bicarbonate/chloride solution at 2000 rpm: (**a**) 4 ppm (**b**) 1 ppm (**c**) 0.8 ppm dissolve oxygen.

**Figure 14:**
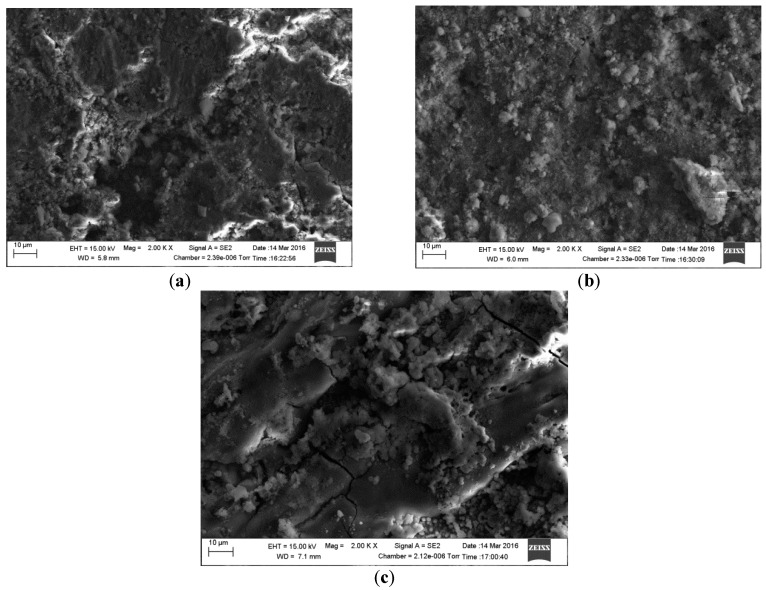
Scanning Electron Microscopy micrographs of mild steel in 5 g/L bicarbonate/chloride solution at 2000 rpm before cleaning: (**a**) 2 h (**b**) 4 h (**c**) 8 h immersion time.

**Figure 15:**
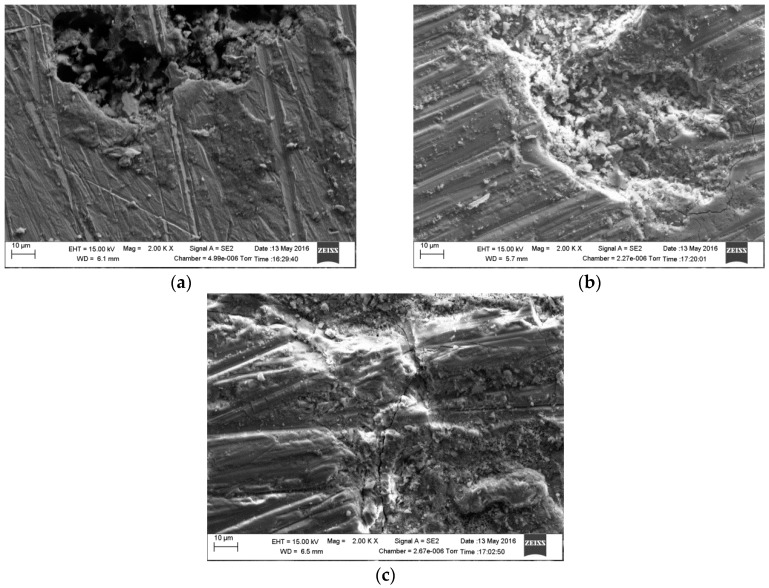
Scanning Electron Microscopy micrographs of mild steel in 5 g/L bicarbonate/chloride solution at 2000 rpm after cleaning: (**a**) 2 h (**b**) 4 h (**c**) 8 h immersion time.

Figure 12 shows scanning electron micrographs obtained from the surfaces of samples immersed in 1 g/L and 5 g/L bicarbonate chloride solutions at 2000 rpm. The SEM results show that pits with relatively small diameters of a few microns were predominant with specimen in low bicarbonate concentration. The density of pits decreased in the sample exposed to 5 g/L bicarbonate solution, but the pits became much larger both in depth and width. Figure 13a–c show the surface morphologies of specimens after corrosion tests in bicarbonate/chloride solution at different dissolved oxygen (DO) concentrations. The micrographs revealed that the corrosion damage decreases with decreasing dissolved oxygen due to more tenacious oxide film on the steel–solution interface, as shown in Figure 13c for solution containing 0.8 ppm DO. Our cross-sectional images (Figure 11) show that the oxide film thickness increases with immersion time, the film morphology also varies with immersion time as shown in Figure 14 providing a barrier layer at the metal–solution interface, and as a result the surface damage also decreases, ranging from pitting corrosion to general corrosion, as shownin Figure 15.

**Figure 16:**
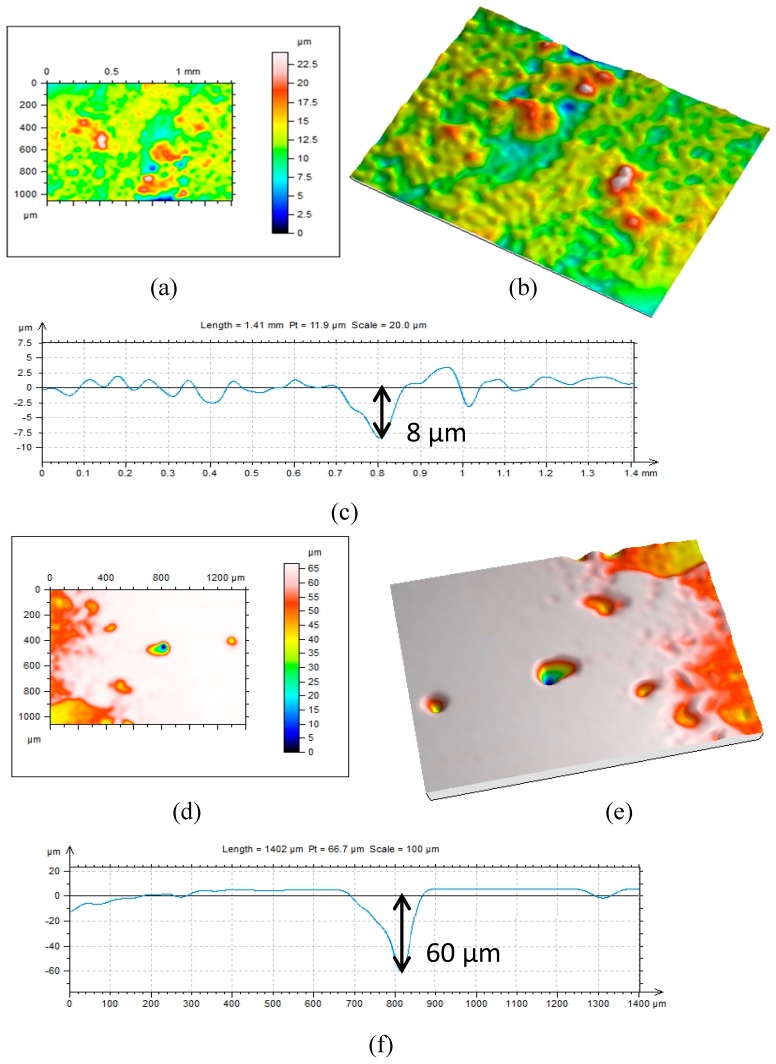
Topography profile and 3D image of pit affected area: (**a**–**c**) 1 g/L and (**d**–**f**) 5 g/L bicarbonate/chloride solution.

The corrosion pit density and pit depth in specimens immersed in 1 g/L and 5 g/L bicarbonate were further studied using Leica optical microscopy. The results show that the corrosion pit density decreases while the depth increases with bicarbonate concentration. The pit depth increases from ≈−8 µm to ≈−60 µm for 1 g/L and 5 g/L, respctively, as shown in Figure 16.

**Figure 17:**
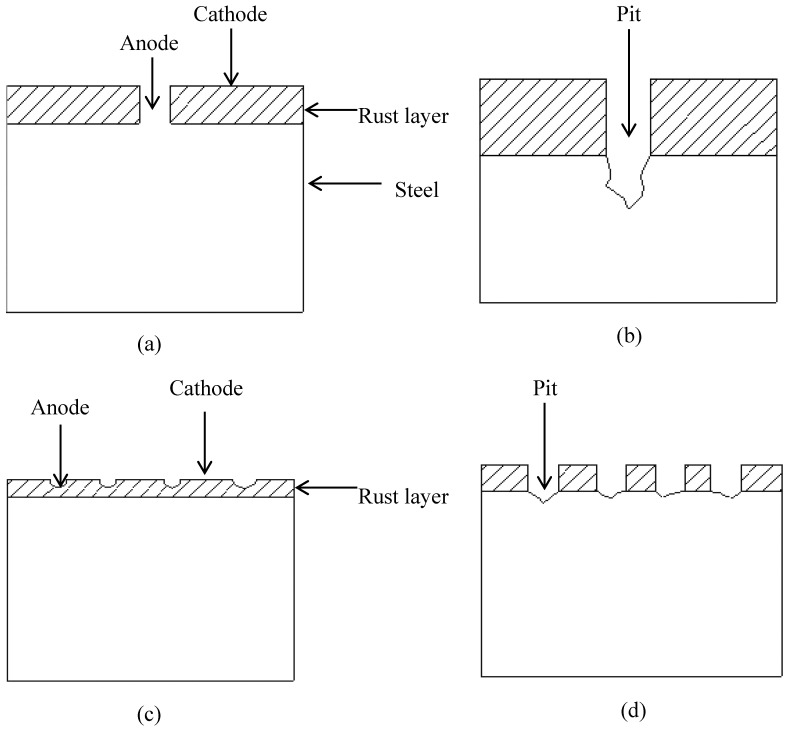
Schematic mechanism of bicarbonate concentration effect: (**a**,**b**) 5 g/L NaHCO_3_ (**c**,**d**) 1 g/L NaHCO_3_ concentration.

The corrosion pit density and pit depth in specimens immersed in 1 g/L and 5 g/L bicarbonate were further studied using Leica optical microscopy. The results show that the corrosion pit density decreases while the depth increases with bicarbonate concentration [36]. The pit depth increases from ≈8 µm to ≈60 µm for 1 g/L and 5 g/L, respctively, as shown in Figure 16. The dissolution of iron is accelerated in the presence of bicarbonate owing to the formation of the stable soluble complex anion Fe (CO_3_)_2_^2−^ as reported in [3]. As more corrosion product is formed at the metal surface, galvanic couples could occur between steel surfaces covered with corrosion products (cathode) and uncovered active site (anode). This unstable thermodynamic condition accelerates the initiation and propagation of localized corrosion as shown in Figure 17.

### 2.5. XRD Analyses

**Figure 18:**
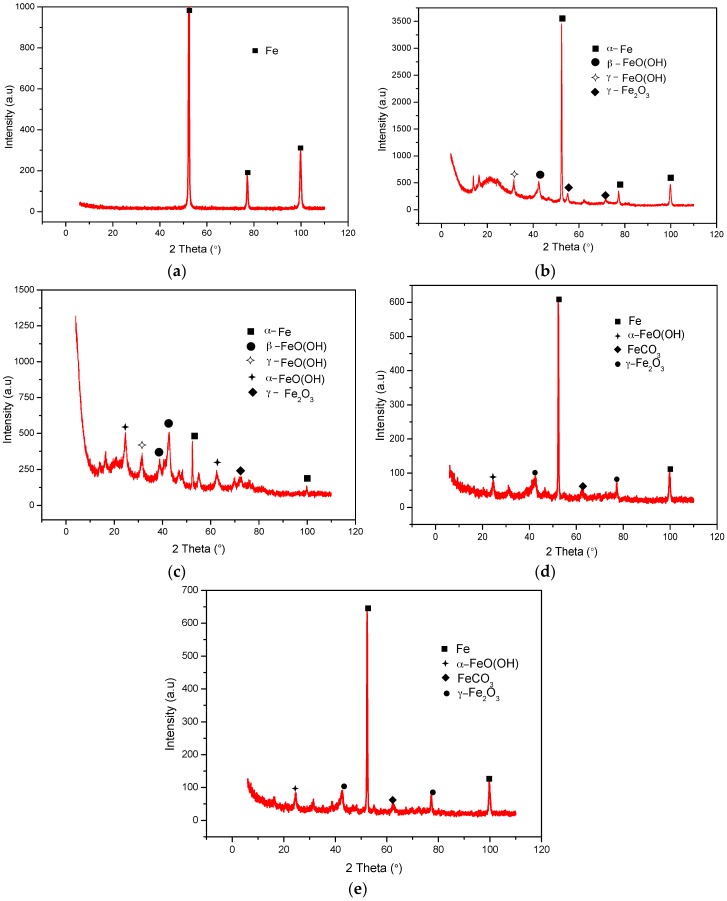
XRD analysis of corrosion products of mild steel in bicarbonate solutions containing chloride at 2000 rpm: (**a**) unimmersed (**b**) 1 g/L and (**c**) 5 g/L bicarbonate (**d**) 4 ppm (**e**) 1 ppm.

Figure 18 shows the XRD patterns for specimens immersed in bicarbonate chloride containing solution at 2000 rpm. Figure 18a–c show the peak patterns for unimmersed and immersed specimens in different bicarbonate concentrations. The elemental composition of the corrosion products tested under different bicarbonate concentrations show similarities in their chemical composition. However, the XRD patterns in Figure 18b indicate diffraction peaks of mainly Fe (iron) (44.3%) and a single peak for β-FeO(OH) (akaganeite) (16.32%) and γ-FeO(OH) (lepidocrocite) (39.34%), in contrast to the peak patterns in Figure 18c, with multiple peaks of both (α-FeO(OH) goethite (58.16%) and β-FeO(OH) (akaganeite) (6.20%). The multiple peaks could probably be due to the density of the corrosion products on the steel surface. The formation of akaganeite confirms the presence of chloride ions in the corrosion products [31,37]. The relatively high amount of akaganeite in the specimen immersed in 1 g·L^−1^ bicarbonate/chloride solution implies that the corrosion product contains more chloride ions. Figure 18d,e show the XRD peak patterns for specimens immersed in 5 g·L^−1^ bicarbonate/chloride containing different dissolved oxygen concentrations. Goethite (α-FeO(OH)), siderite FeCO_3_ and maghemite (γ-Fe_2_O_3_) were formed at each DO concentration. However, more siderite FeCO_3_ and maghemite (γ-Fe_2_O_3_) peaks are evident as DO decreases, as shown in Figure 18e.

### 2.6. XPS Analyses

**Figure 19:**
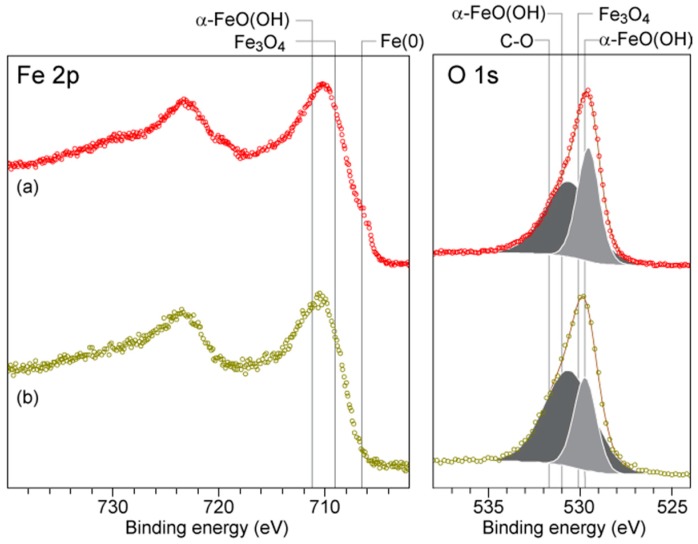
XPS collected from the Fe 2p (left) and O 1s (right) regions of Samples: (**a**) 1 g/L and (**b**) 5 g/L. The Fe 2p data are presented without background subtraction, and without deconvolution of the peaks. The O 1s data have had a Shirley background subtracted and have been deconvolved into two contributions. The indicated positions for different chemical states are approximate, and are based on values taken from [38,39].

Prior to XPS measurements, samples were sputtered for 60 min using 2 keV Ar^+^ ions to remove adsorbed surface contamination and reveal the underlying chemistry in more detail. Argon sputtering is known to convert FeCO_3_ to FeO [38], and, correspondingly, we did not observe FeCO_3_, which can be identified from its high-binding-energy contribution to C 1s, in our spectra. All spectra were calibrated to the C-C adventitious carbon peak at 284.8 eV. In each spectrum, the dominant C 1s component was identified as adventitious C-C. In each case, this peak had the expected companion C-O/C=O peaks at higher binding energy. The O 1s spectra of the two samples (Figure 19) were similar, and could be deconvolved into two peaks: a relatively sharp peak near 530 eV (dark gray component) and a broader peak centred near 531 eV (light gray component). The lower binding energy peak has the expected shape and location for O^2−^ in iron oxides (Fe_3_O_4_, α-FeO(OH) and FeO). The broader, higher binding energy peak is consistent with the OH^−^ oxygens in α-FeO(OH), as well as with the C-O contribution from adventitious carbon. The analysis of Fe 2p is complicated due to the multiplet structure of Fe 2p peaks for high-spin compounds comprising Fe^2+^ and Fe^3+^ [39]. The multiplet structure manifests in a broad, asymmetric peak shape, particularly when measured with unmonochromated radiation as in the present measurements, making deconvolution of contributions from multiple chemical states difficult, and hence we offer only a qualitative analysis of the Fe 2p spectra shown in Figure 19. As with O 1s, the samples show similar peak shapes. The spectral weight in each sample is centred near 710 eV, consistent with contributions from Fe_3_O_4_ and α-FeO(OH).

## 3. Experimental Section

### 3.1. Materials and Solutions

Cylindrical carbon steel rod (∅ 16 mm) of grade AISI 1020 was utilized as the rotating disc electrode (RDE). The chemical composition of the as-received rod was analyzed using a Field Emission Electron Probe Microanalyzer (JXA-8530F) with results shown in Table 5. The test samples were polished using emery papers of grit sizes ranging from 220 to 800. Two solutions were prepared by dissolving analytical grade sodium bicarbonate (1 g, 5 g) and 2 g of sodium chloride in 1 L of distilled deionized water. The solutions were deaerated by bubbling nitrogen gas three hours before sample immersion to study the effect of bicarbonate concentrations and immersion time. To study the effects of dissolved oxygen, nitrogen gas was bubbled for 0 h, 2 h and 3 h which resulted in 4 ppm, 1 ppm and 0.8 ppm DO concentrations, respectively, before sample immersion. The electrochemical tests were performed at ambient temperature (22 ± 3 °C) at pH (8.2 ± 0.1).

### 3.2. Electrochemical Test

The corrosion tests were carried out in a 1 L glass cell containing the rotating disk electrode (working electrode) fitted into rotating shaft of an analytical rotator AFASRE 747 (Pine Instrument). The reference electrode was a saturated calomel electrode (SCE) connected to the cell by a bridge and a Lugging capillary Platinum was used as counter electrode. The angular speed (ω) of the rotating disc electrode was controlled by the ASR RDE speed controller. The rotation speed of the RDE performed in the tests was 2000 rev/min, respectively. Corrosion evaluation was monitored by potentiodynamic polarization and electrochemical impedance spectroscopy (EIS). The tests were performed at a scan rate of (0.167 mV/s) using a Bio-Logic instrument Model VSP 0508 potentiostat. The potentiodynamic polarisation was obtained from a starting potential of −0.30 V vs. SCE to a final potential of +1.20 V vs. SCE. Electrochemical impedance spectroscopy measurements were conducted at open-circuit. Measuring frequencies ranged from 10 kHz to 0.1 Hz with a perturbing alternating current (AC) amplitude of 10 mV and a sampling rate of 10 points per decade.

### 3.3. Surface Analysis

Scanning electron microscopy (SEM) imaging was performed after corrosion tests using a Zeiss Sigma VP Field Emission Scanning Electron Microscope (Oxford XMax 50 silicon drift energy dispersive spectroscopy EDS detector) in secondary electron (SE) image mode with an accelerating beam voltage of 15 kV. Pit volume and pit depth were examined using Leica Optical microscopy Model DFC 490.

### 3.4. X-ray Diffraction

The corrosion products were characterized in-situ using a PANalytical X’pert PRO MPD powder X-ray Diffractometer (XRD) equipped with a 40 keV, 40 mA, Co K_α_. A parabolic mirror optic on the incident side, 0.04 rad Soller slits on the source and detector side, and 0.09° collimator before the detectors were used to give a parallel beam. The incident angle (ω) was fixed to 3° and data collected from 6° to 90° (2θ). The other slits were optimized to ensure the beam footprint did not exceed the sample dimensions or the detector window opening. Quantitative phase analysis was performed in TOPAS (V5, Bruker). An instrument function collected from SRM 660a was used to accurately model peak shape and width. Fixed incident parallel beam intensity and peak width corrections according to Rowles and Madsen [40], Toraya et al. [41] and Haggerty et al. [42] were employed. The values of these corrections were fixed to those for the instrument function when refining the sample data. Other parameters refined included background, scale factors for each phase, specimen displacement, unit cell parameters for each phase, and a Lorentzian crystallite size term for each phase.

### 3.5. X-ray Photoelectron Spectroscopy

XPS measurements were performed using a non-monochromatized Al Kα (1486.7 eV) source (DAR 400, ScientaOmicron GmbH) with a 125 mm hemispherical electron energy analyser (Sphera II, 7 channel detector, ScientaOmicron GmbH). The XPS was housed in an ultrahigh vacuum chamber with a base pressure of 10^−11^ mbar. XPS data were collected over Fe, C and O core levels at a pass energy of 20 eV using 0.1 eV steps and a dwell time of 0.1 s. The O 1s core level was fit using symmetric components after subtracting a Shirley background.

## 4. Conclusions

The electrochemical behavior of mild steel in different HCO_3_^−^, DO concentrations and immersion time was studied, using potentiodynamic polarization and electrochemical impedance spectroscopy. Pitting corrosion depth increases with increasing bicarbonate concentrations. Lowering the amount of dissolved oxygen facilitates passivation, whereas an increase in dissolved oxygen and HCO_3_^−^ in bicarbonate/chloride results in a significant increase in the cathodic oxygen reduction process and anodic metal dissolution due to oxidation. The corrosion current density decreases significantly with decreasing DO concentration, dropping from 201.5 µA·cm^−2^ at 4 ppm to 22.3 µA·cm^−2^ at 0.8 ppm. A relatively stable oxide films were formed at ~600 mV_SCE_ at lowered DO (1–0.8 ppm) concentrations. Immersion time has an important effect: the oxide film thickness increases and the corrosion rate decreases with increasing immersion time. Internal corrosion of mild steel pipelines in high bicarbonate/chloride environment can be minimized when DO concentration is relatively low at pH ≥ 8 and potential ≈ −600 mV_SCE_. Oxygen is necessary for passive film formation, but can be detrimental to mild steel above the critical level in high bicarbonate/chloride solution under dynamic conditions.

## Figures and Tables

**Table 1:** Polarization parameters for mild steel in 5 g/L bicarbonate/chloride solution in different dissolved oxygen concentrations.

*DO* (ppm)	I_corr_ (µA·cm^−2^)	E_corr_ (mV)	b_a_ (mV·dec^−1^)	−b_c_ (mV·dec^−1^)
0.8	22.28	−767.5	163.0	302.9
1	25.24	−780.6	138.5	222.9
4	201.52	−369.2	243.5	240.3

**Table 2:** Impedance parameters for mild steel in bicarbonate/chloride solution in different dissolved oxygen concentrations.

*DO* (ppm)	*R_s_* (Ω·cm^2^)	*R_ct_* (Ω·cm^2^)	*Q_dl_* (F·cm^−2^)	*n*	*R_a_* (Ω·cm^2^)	*Q_a_* (F·cm^−2^)
0.8	46.3	1015	1.76 × 10^−3^	0.77	440.04	5.9 × 10^−4^
1	53.8	874.7	1.18 × 10^−3^	0.66	90.49	1.39 × 10^−3^
4	63.5	104.7	6.88 × 10^−3^	0.59	33.04	3.75 × 10^−3^

**Table 3:** Polarization parameters for mild steel in 5 g/L bicarbonate/chloride solution for different immersion times at 2000 rpm.

Immersion Time (h)	I_corr_ (µA·cm^−2^)	E_b_ (mV)	E_corr_ (mV)	b_a_ (mV·dec^−1^)	−b_c_ (mV·dec^−1^)
2	22.28	−355.0	−767.5	163.0	302.9
4	8.98	−292.3	−820.4	126.6	138.0
8	6.04	−229.1	−833.9	145.6	134.8

**Table 4:** Impedance parameters for mild steel in bicarbonate/chloride solution for different immersion times.

Immersion Time (h)	*R_s_* (Ω·cm^2^)	*R_ct_* (Ω·cm^2^)	*Q_dl_* (F·cm^−2^)	*n*	*R_a_* (Ω·cm^2^)	*Q_a_* (F·cm^−2^)
2	46.3	1015	1.76 × 10^−3^	0.77	440.04	5.9 × 10^−4^
4	30.5	1370	1.65 × 10^−3^	1	804.0	5.04 × 10^−4^
8	41.6	2700	7.28 × 10^−4^	0.82	618.6	5.83 × 10^−4^

**Table 5:** Chemical composition of mild steel *(wt %)*.

C	Si	Mn	Cr	Cu	Ni	Fe
0.20	0.32	0.79	0.01	0.01	0.01	Bal
